# Loneliness and the degree of addiction to shopping and work among Polish women: the mediating role of depression

**DOI:** 10.1192/j.eurpsy.2023.1381

**Published:** 2023-07-19

**Authors:** K. Rachubińska, A. M. Cybulska, D. Schneider-Matyka, E. Grochans, S. Grochans

**Affiliations:** 1 Department of Nursing; 2 Department of Clinical Nursing, Pomeranian Medical University in Szczecin, Szczecin, Poland

## Abstract

**Introduction:**

The rapid development of civilization is accompanied by many changes affecting human functioning. Technological development, wide access to positive stimuli and the neglect of emotional control and self-awareness in the learning process make it difficult for individuals to control their behavior. Such a loss of control is associated with the development of addictions. Therapists, doctors and researchers more and more often encounter cases of compulsive behavior focused on a specific activity - apart from gambling or playing computer or internet games, more and more often the subjects of preoccupation are compulsive shopping, sexual activities and work (workaholism).

**Objectives:**

The aim of the research was to try to define the mediating role of depression in the relationship between addiction to shopping and work and loneliness, understood in terms of general loneliness among Polish women.

**Methods:**

The study was conducted among 556 women. The research was carried out with the use of the diagnostic survey method, using the questionnaire technique: the De Jong Gierveld Loneliness Scale, the Purchasing Behavior Scale, the Work Addiction Risk Test, Beck Depression Inventory and a questionnaire of our authorship.

**Results:**

Depressiveness is a mediator in the relationship between the feeling of loneliness and the degree of addiction to shopping (β = -0.0246, z = -2.03, p = 0.043) and in the relationship between the feeling of loneliness and the degree of addiction to work (β = -0.0722, z = -4.002, p < 0.001). The direct impact of the feeling of loneliness on the degree of addiction to shopping (p = 0.237) and work (p = 0.576) is statistically insignificant.

**Conclusions:**

In the mediation model adopted, it was shown that depressiveness plays the role of a mediator between the feeling of loneliness and the degree of addiction to shopping and work. The increase in the level of depression increased the degree of addiction to shopping and work. The mediator’s participation lowered the level of the feeling of loneliness. Loneliness was not a significant predictor of addiction to shopping and work. There is a need to include activities aimed at identifying psychological factors influencing the occurrence of addictions to shopping and work among women. It seems important to be able to use psychological help when needed. It is also necessary to take institutional preventive measures to prevent the occurrence of behavioral addictions among women.

**Disclosure of Interest:**

None Declared

